# Ocean acidification disrupts the orientation of postlarval Caribbean spiny lobsters

**DOI:** 10.1038/s41598-020-75021-9

**Published:** 2020-10-22

**Authors:** Philip M. Gravinese, Heather N. Page, Casey B. Butler, Angelo Jason Spadaro, Clay Hewett, Megan Considine, David Lankes, Samantha Fisher

**Affiliations:** 1 Mote Marine Laboratory and Aquarium, Fisheries Ecology and Enhancement Program, Sarasota, FL 34236 USA; 2 Department of Biological Sciences, Florida Southern College, Lakeland, FL 33801 USA; 3 Mote Marine Laboratory and Aquarium, Elizabeth Moore International Center for Coral Reef Research and Restoration, Summerland Key, FL 33042 USA; 4 Sea Education Association, Woods Hole, MA 02543 USA; 5 Fish and Wildlife Research Institute, Florida Fish and Wildlife Conservation Commission, Marathon, FL 33050 USA; 6 Department of Marine Science and Technology, The College of the Florida Keys, Key West, FL 33042 USA

**Keywords:** Marine biology, Climate-change impacts, Behavioural ecology

## Abstract

Anthropogenic inputs into coastal ecosystems are causing more frequent environmental fluctuations and reducing seawater pH. One such ecosystem is Florida Bay, an important nursery for the Caribbean spiny lobster, *Panulirus argus*. Although adult crustaceans are often resilient to reduced seawater pH, earlier ontogenetic stages can be physiologically limited in their tolerance to ocean acidification on shorter time scales. We used a Y-maze chamber to test whether reduced-pH seawater altered the orientation of spiny lobster pueruli toward chemical cues produced by *Laurencia* spp. macroalgae, a known settlement cue for the species. We tested the hypothesis that pueruli conditioned in reduced-pH seawater would be less responsive to *Laurencia* spp. chemical cues than pueruli in ambient-pH seawater by comparing the proportion of individuals that moved to the cue side of the chamber with the proportion that moved to the side with no cue. We also recorded the amount of time (sec) before a response was observed. Pueruli conditioned in reduced-pH seawater were less responsive and failed to select the *Laurencia* cue. Our results suggest that episodic acidification of coastal waters might limit the ability of pueruli to locate settlement habitats, increasing postsettlement mortality.

## Introduction

In nearshore and shallow coastal waters, seawater pH varies daily and seasonally, often more strongly than in the open ocean because of the smaller water volumes involved^1–3^. The variation in coastal seawater pH is also driven by a number of processes that can occur simultaneously with daily and seasonal fluctuations including benthic biological activity, storms, tidal cycles, and seasonal changes in biogeochemical processes^4–8^. Anthropogenic factors such as increased carbon dioxide emissions, increased nutrient-rich runoff, eutrophication, and changes in land use can amplify the acidification of coastal waters in both the short and long term^2,9,10^. These reductions in seawater pH can adversely affect coastal species, especially during early ontogenetic stages, which can be more sensitive to fluctuating environmental conditions^11–14^.

Among marine crustaceans, earlier life stages (embryos, larvae, and juveniles) are often more sensitive to environmental extremes, primarily because they are still developing the physiological mechanisms needed to adjust to environmental fluctuations^8^. For example, embryos of the green porcelain crab, *Petrolisthes cinctipes,* were reported to have slower metabolic rates and reduced heart rates during exposure to reduced-pH seawater, while the metabolic rates of adults were unaffected^15,16^. Additionally, the survival of juvenile *P. cinctipes* was also significantly reduced under reduced pH exposure^16^. Larvae of the blue crab, *Callinectes sapidus,* were 10% smaller and experienced a 25% reduction in survival when raised in reduced-pH seawater^17^; adult blue crabs, however, were more resilient to ocean acidification and increased calcification in reduced-pH seawater^18^. Although these studies demonstrate the sensitivity of early crustacean life stages to reduced-pH conditions in terms of survival, metabolism, and growth, less is known about the consequences of ocean acidification on the behavioural responses of crustaceans^19^.

Many marine organisms modify their behavioural responses to exogenous stimuli to facilitate navigation and orientation in their environment^13,14,20–24^. Among crustaceans, chemosensory cues are important stimuli used throughout ontogeny for a variety of purposes including orientation, identification of specific settlement habitats, predator avoidance, locating prey and foraging, and interacting with conspecifics^20–24^. Recently, several studies have demonstrated that reductions in seawater pH can disrupt an individual’s chemosensory acuity^23–29^. For example, reduced seawater pH can change not only a cue’s chemical structure^28,29^ but also an animal’s ability to identify the cue and respond to it with an appropriate behavioural response^30,31^. Regardless of the nature of the chemosensory disruption, changes in a species’ behavioural responses during exposure to reduced pH can affect settlement and recruitment processes, especially if they increase the risk of predation.

The Caribbean spiny lobster, *Panulirus argus,* relies upon chemical cues throughout ontogeny^32–37^. Postlarval *P. argus* use chemical cues, like compounds released by the red macroalgae *Laurencia* spp., to identify appropriate settlement habitats^32^. Juvenile spiny lobsters use olfactory cues to identify and aggregate with conspecifics^33–35^. Olfactory cues can also play a role in the Caribbean spiny lobster’s social quarantine behaviours, as individuals can identify and avoid conspecifics infected with *P. argus* virus 1 (PaV1), a lethal species-specific pathogen^36,37^. In fact, recent work suggests that reduced seawater pH may impair the ability of juvenile *P. argus* to detect the odour of, and to differentiate between, healthy and diseased conspecifics, possibly negating their social quarantining and increasing rates of disease transmission^27^. How reduced seawater pH affects chemosensory behaviour in *P. argus* postlarvae is not known but is of critical importance, given the sensitivity of *P. argus* to chemosensory stimuli and the behavioural impairment reported for juvenile *P. argus* under acidified conditions^27^.

After nine months of larval development and dispersal offshore, larval Caribbean spiny lobsters metamorphose into puerulus postlarvae (hereafter referred to as pueruli) and migrate shoreward, where they begin to seek out settlement habitats^32^. Florida Bay is a shallow-water habitat that stretches between the southern end of the Florida Peninsula and the Florida Keys and serves as a critical nursery habitat for *P. argus*^37,38^. The shallow nature of Florida Bay can restrict flow in some habitats, while its proximity to land results in seasonal outflows that can subject Florida Bay to extreme fluctuations in environmental conditions, including seawater pH^39–41^. We therefore investigated whether reduced seawater pH results in modification of the chemosensory behaviour of *P. argus* postlarvae. Specifically, we tested how pueruli, conditioned in reduced-pH seawater, responded to chemical odours released by the red macroalgae *Laurencia* spp., a known settlement-habitat cue using a Y-maze choice chamber^32,42–44^. During each experimental trial we compared several behavioural responses that included: the number of pueruli that moved to the cue side of the chamber with the number of pueruli that moved to the side with no cue as well as pueruli response times (sec). Spiny lobsters support the most valuable fishery throughout Florida and the Caribbean, worth nearly US$500 million annually^45^. Therefore, identifying how shifts in environmental conditions, like seawater acidity, might affect the behaviour and recruitment of *P. argus* pueruli into Florida Bay and the Florida Keys will help inform fishery managers as to the risks (e.g., recruitment bottlenecks) associated with coastal acidification.

## Results

### Response to chemical cues

**Figure 1:**
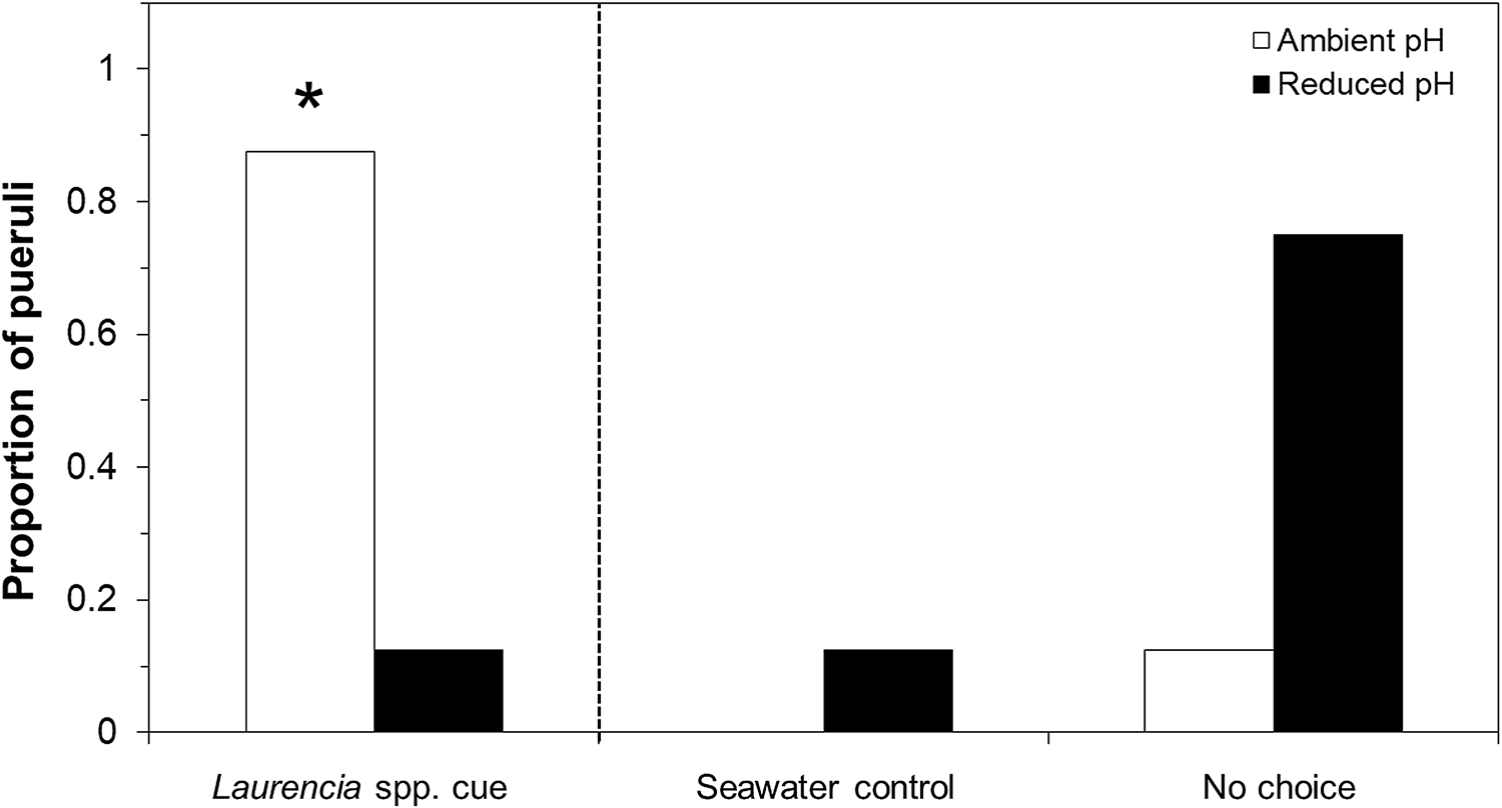
The proportion of pueruli that selected the *Laurencia* spp. cue side of the chamber between treatments (left panel). The asterisk above the bar in the left panel indicates a significant difference at the α = 0.05 level using a binomial test. The right panel shows the proportion of animals that selected the seawater (no cue) side of the chamber and the proportion of animals that displayed no choice.

Pueruli conditioned in ambient-pH (control) seawater selected the *Laurencia* spp. cue side of the chamber 87.5% of the time (Fig. 1). Only 12.5% of pueruli conditioned in reduced-pH conditions selected the *Laurencia* spp. cue side of the chamber, which was significantly different from the control (binomial test: z =  − 3.10, *p* = 0.001, n = 16 individuals per treatment condition; Fig. 1). Of the pueruli conditioned in ambient-pH seawater that entered the Y-section of the chamber (n = 14), 100% oriented toward the *Laurencia* spp. cue side. Conversely, only 2 (12.5%) pueruli conditioned in the reduced-pH seawater selected the *Laurencia* spp. cue side of the chamber, while 2 (12.5%) other pueruli conditioned in reduced-pH seawater selected the side of the chamber with no chemical cue. A G-test showed that the proportion of ambient-pH pueruli entering the *Laurencia* spp. cue side of the Y-maze indeed differed significantly from a 50–50 distribution (G = 63.5, P < 0.001).

### Pueruli activity in the choice chamber

**Figure 2:**
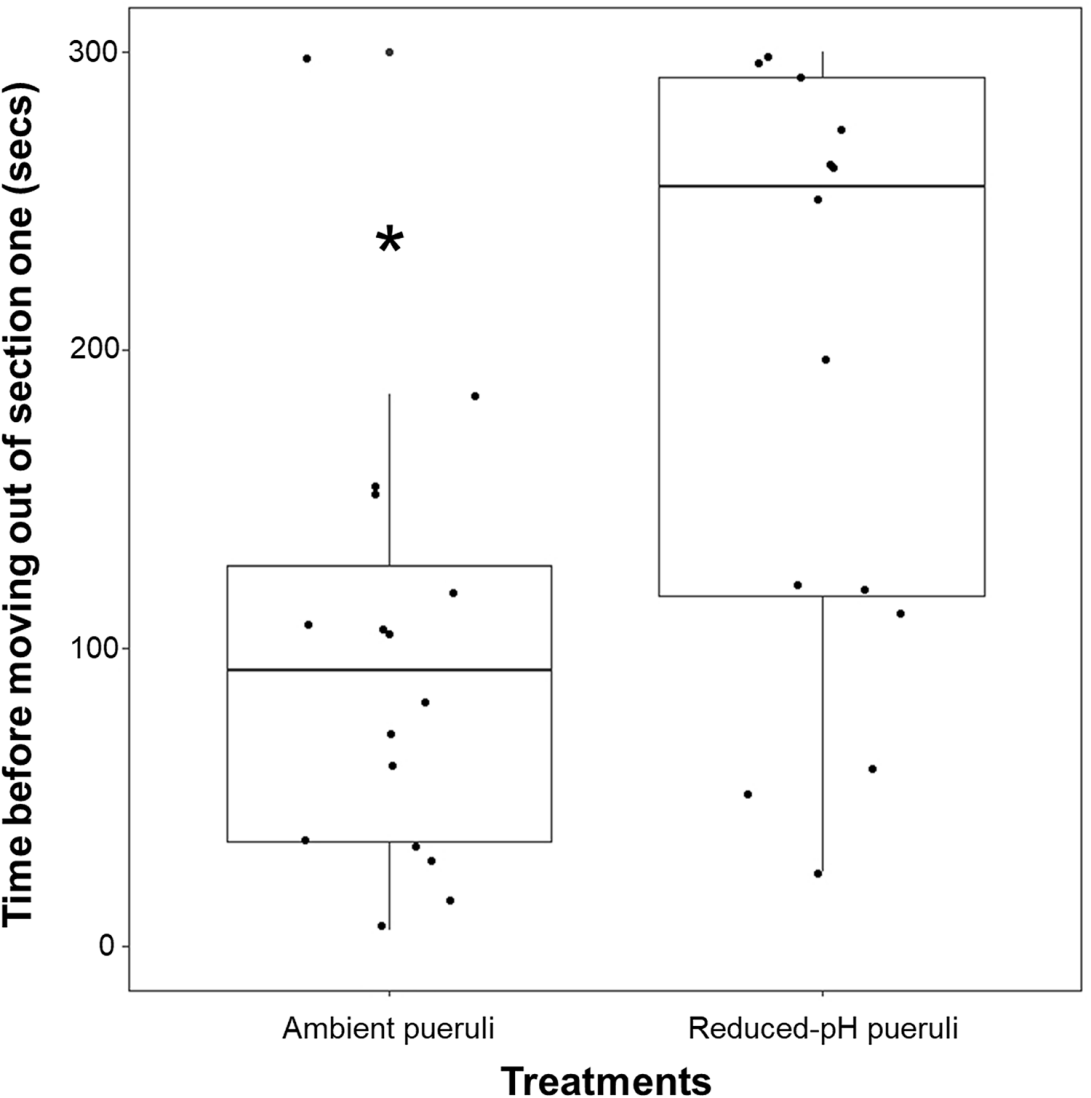
Box plot of the time (sec) before pueruli moved out of section one of the chamber between treatments. The asterisk indicates a significant difference at the α = 0.05 level as indicated by a Wilcoxon rank sum test (W = 58.5, df = 1, *p* = 0.009). The boxes represent the first and third quartile, the horizontal lines within the boxes represent the median, and the whiskers represent the range of the data. The points represent each of the n = 16 individual’s response within each treatment.

Pueruli conditioned in the reduced-pH treatment were significantly slower to move out of section one of the chamber, as evidenced by a longer response time before searching for the *Laurencia* spp. cue than individuals in the ambient-pH treatment (W = 58.5, df = 1, *p* = 0.009, n = 16 individuals per treatment condition; Fig. 2). Pueruli conditioned in the reduced-pH treatment remained inactive in section one for a median of 255 s ± 45 MAD before they began searching or exploring the chamber, whereas animals in the control were inactive for a median of only 92 s ± 57.5 before they began searching or exploring. We observed no mortality in either treatment during experimentation.

## Discussion

Florida Bay, like many coastal habitats, is experiencing unfavorable or inimical conditions, which can shift environmental conditions beyond the tolerance of some species, especially during their early, more sensitive life stages^2–4,12,14,46–50^. In autumn, when spiny lobster pueruli are settling, the seawater pH in parts of Florida Bay can experience a seasonal change, becoming more acidic over short time scales^4^. These shifts in pH and *p*CO_2_ may become more extreme with the changes forecasted with anthropogenic climate change^10^, and may result in adverse effects on the *P. argus* fishery, especially since Florida Bay serves as one of the most important nursery habitats for spiny lobster settlement and recruitment^32^. Despite the importance of this habitat to *P. argus* and the fishery it supports, only one study has described how changes in coastal seawater pH might affect the processes that mediate spiny lobster settlement^27^. Here we demonstrate that after exposure to reduced-pH seawater conditions, spiny lobster pueruli may not respond to a known settlement cue like odours emitted by *Laurencia* spp. macroalgae, which is associated with advantageous settlement habitats for the species^32,42–44^.

Spiny lobster pueruli conditioned in reduced-pH seawater were less responsive to *Laurencia* spp. chemical cues than pueruli conditioned in control pH seawater. This is reflected in the observations in which pueruli conditioned in reduced-pH took longer to move out of the first section of the chamber before actively moving into other sections (Fig. 2). Animals exhibiting a stress or “shock” response to changing pH would likely alter their behavior by reduced swimming and movement in general^29^. Most animals conditioned in reduced pH in our experiments were still observed moving through the chamber (13 out of 16 animals) and we observed no mortality in either treatment suggesting that reduced-pH was likely inhibiting the pueruli’s ability to sense the cue, however, we cannot exclude the possibility that shock was a contributing factor to the reduced-pH pueruli response. However, since pH can have a wide range of variability in small pockets of seawater^46^, especially in nearshore eutrophic waters^2,6,47^, the responses identified by pueruli in our study should be similar to those that occur in situ, even if those are shock-type responses. The chemosensory abilities of the Caribbean spiny lobster have been well documented, and studies over the past several decades have repeatedly demonstrated the importance of chemical sensing throughout ontogeny^32–37,43,44^. Here we demonstrate that rapid reductions in seawater pH (decreases by 0.3 pH units) similar to diel fluctuations reported in other coastal habitats (e.g., Tampa Bay monitoring station: https://tampabay.loboviz.com/) may temporarily reduce puerulus activity and that individuals subjected to rapid reductions in pH could have difficulty in sensing the *Laurencia* spp. chemical cues that would otherwise be preferred at settlement^32^. Our results align with recent work by Ross and Behringer^27^, who also demonstrated chemosensory impairment of juvenile *P. argus* under reduced-pH conditions. In their study, juvenile spiny lobsters did not display attraction behaviours toward shelters with conspecific cues when under reduced-pH conditions.

Behavioural impairment under reduced-pH seawater conditions is likely species-specific and may vary during ontogeny in crustaceans^12,14,16–18^. For example, stage III larval stone crabs (*Menippe mercenaria*) swam upwards during exposure to ambient pH seawater conditions, but reversed their swimming direction by swimming downwards and at a faster rate when exposed to reduced seawater pH, while there was no observable change in the directional movement of later larval stone crab instars^14^. The adults of some marine invertebrate species also display difficulty in their ability to detect exogenous stimuli or experience depressed activity levels after exposure to reduced-pH conditions. For instance, while exposed to reduced-pH conditions, adult hermit crabs failed to locate prey and were less active^48^, and adult shrimp reduced their swimming activity^49^. These studies demonstrate that a reduction in seawater pH can disrupt responsiveness to important sensory cues and reduce an individual’s activity level, both of which can decrease survivorship and contribute to declining populations during periodic or extreme reductions in seawater pH^14^.

It was not an objective of the present study to identify the physiological mechanisms of the altered behaviours and decreased activity among spiny lobster pueruli conditioned in reduced-pH conditions, but other experiments have suggested that when crustaceans are exposed to acidified seawater, the pH of their hemolymph may decrease, which can temporarily disrupt ion regulation, alter enzyme structure and activity, and impair metabolism, at least until homeostasis is restored^50–52^. Alternatively, some species may also experience temporary alkalosis during hemolymph maintenance or compensation as the internal *p*CO_2_ would decrease and the extra bicarbonate in the hemolymph would temporarily increase pH above the level of homeostasis^53^. Temporary alkalosis impairment would likely take several hours depending on the amount of time needed for the animal to reduce the excess bicarbonate; however, our experimental trials were on the magnitude of minutes. Inability to regulate acid–base balance in acidified seawater may therefore result in physiological changes that alter an individual’s metabolic demand and so decreases the animal’s ability to actively search for or respond to a chemical cue^50^. Such physiological changes can modify an individual’s chemosensory acuity by slowing antennular flicking in crustaceans, including juvenile spiny lobsters, a behaviour that ensures that chemical cues reach the olfactory system^27,54–58^. Although we did not directly quantify antennular flicking in our experiments, we did observe less antennular movement among individuals conditioned in the reduced-pH treatment. Chemoreceptors are also located on the antennules of the pueruli and exposure to reduced-pH seawater may damage these receptors^27,54–56^. Damaged chemoreceptors could prevent the individual from recognizing a chemical cue and might help explain why pueruli from the reduced-pH treatment in our study were less successful in detecting *Laurencia* spp. cues^27,54–56^. If the chemosensory receptors are damaged, a cue may need to be more concentrated than the one we used in these experiments for a behavioural response to be observed. Our cue concentration, however, was within the range of those used in other chemosensory studies on spiny lobsters^59,60^. Overall, the decrease in activity observed among individuals supports our hypothesis that some physiological mechanism is likely impaired in spiny lobster pueruli during exposure to reduced-pH conditions. Regardless of the physiological mechanism, the high levels of inactivity and lethargy among pueruli exposed to reduced-pH could increase mortality.

Spiny lobster pueruli have been shown to detect coastal chemosensory cues in seawater collected as far as 30 km offshore of the Florida reef tract, thus providing a habitat-specific directional stimulus over large spatial scales^32^. The movement of spiny lobster pueruli toward Florida Bay requires that individuals move across shallow seagrass and hard-bottom habitats in which *Laurencia* spp. is known to be abundant^42,61^. Detection of positive settlement cues, like *Laurencia* spp., is advantageous for a species in locating habitats that both are less prone to predation and are more likely to have suitable prey available once pueruli have metamorphosed into the juvenile stage^60^. An inability to identify positive settlement habitats and a decrease in an individual’s activity level when experiencing acidified seawater would make lobster pueruli more susceptible to predation. This could reduce the number of juvenile lobsters entering the fishery, especially if anthropogenic inputs continue to acidify coastal waters (e.g., RCP 8.5)^10^. Postsettlement mortality in spiny lobsters is estimated to range between 97 and 99%^61–63^. Inactivity resulting from exposure to reduced-pH conditions could, therefore, increase this mortality and be detrimental to fishery production.

Acidified seawater conditions may not be the only environmental condition affecting recruitment of pueruli into Florida Bay and the Florida Keys. The observed effects of reduced pH on the ability of pueruli to identify chemosensory stimuli may be compounded in shallow coastal habitats as seawater temperature and salinity, which also affect the chemosensory function of pueruli and juvenile spiny lobsters^27,32^, are both expected to increase in Florida Bay as reduced freshwater delivery and increases in both temperature and sea level are projected to continue with climate change^64–67^. Future studies, therefore, should aim to identify the effects of a suite of stressors on the ability of spiny lobster pueruli to identify ecologically important habitat cues.

## Methods

### Animal collection

In the Florida Keys, Caribbean spiny lobster pueruli settle in nearshore habitats during the new moon with a pronounced maximum during the spring and autumn^60–63^. In this study, pueruli were collected using subsurface plankton tows during spring (March–April) and autumn (October) of 2019. Plankton tows were performed near Duck Key, Florida, USA (24.778716–80.913902) by suspending a plankton net (Seagear, 0.5 m diameter × 1.5 m length; 500-µm mesh) from the Tom’s Harbor Cut bridge. Tows were performed before and after the new moon during the nocturnal flood tide.

### Animal maintenance in the experimental system

Pueruli collected during the plankton tows were transported to Mote Marine Laboratory’s Elizabeth Moore International Center for Coral Reef Restoration and Research, on Summerland Key, Florida, and were maintained in the Climate and Acidification Ocean Simulator (CAOS), an outdoor experimental facility that contains flow-through aquaria in temperature-controlled raceways. The CAOS facility pumps natural seawater from the Atlantic side of the Florida Keys through both a sand filter and a 20 µm particle filter before the seawater enters 3800-L header tanks in which pH is manipulated by injection of CO_2_ using high-precision dosing apparatus (Walchem controllers that regulate Venturi pumps and solenoids). This dosing system records pH every 2 s and will trigger CO_2_ manipulation within ± 0.01 of the target pH value. Ambient seawater pH (control) was maintained at (7.96 ± 0.03 [mean ± SD]), which is within the range of pH values recorded for Florida Bay^4^. The experimental reduced-pH treatment was maintained at (7.62 ± 0.02), which mimics pH conditions projected for 2100 under the Intergovernmental Panel on Climate Change’s business-as-usual CO_2_-emission scenarios (Representative concentration pathway 8.5) and is within the range of episodic diel fluctuations in pH experienced by nearshore habitats^10,68,69^.

Pueruli were randomly assigned to either the control (ambient-pH) or the reduced-pH treatment and immediately placed into the experimental aquaria where they were conditioned in their respective treatment water and maintained separately by collection date and treatment for 72 h before experimentation. Pueruli generally began molting into the early benthic juvenile stage 4–6 days after collection from the plankton. The acclimation aquaria (3.8 L) were held in a flow-through raceway (flow rates for control: 31.1 ml s^−1^, ± 0.83 SD; flow rates for reduced-pH: 30.6 ml s^−1^, ± 0.71 SD) and positioned so that each received its respective treatment seawater. The COAS system is an outdoor facility and so animals were held on a natural photoperiod. During this acclimation period, seawater temperature, salinity, and pH were monitored daily in the aquaria and header tanks using a handheld YSI Professional Plus multiprobe instrument (Texas Instruments Inc.). Probes used to monitor dissolved oxygen and seawater pH were calibrated daily using a one-point 100% air saturation and three-point calibration with NBS buffers, respectively. Sampling and analysis of carbonate chemistry measurements were made according to best practices for seawater CO_2_ measurements^70^. To characterize the carbonate chemistry of the experimental system, weekly seawater samples for laboratory analyses of dissolved inorganic carbon (DIC) and total alkalinity (TA) were collected in 125-mL borosilicate glass bottles and immediately poisoned with 60 μL of saturated mercuric chloride (HgCl_2_). Dissolved inorganic carbon was determined using a DIC analyser (Apollo SciTech), and TA was determined using an automated titrator (Metrohm AG, Model 905 Titrando); accuracy and precision of the instruments were tested using certified reference materials for seawater CO_2_ from the Dickson Laboratory (Scripps Institution of Oceanography, UCSD). Seawater temperature, salinity, DIC, and TA were used to constrain carbonate chemistry parameters using the *seacarb* package in R^70,71^. K1 and K2 dissociation constants from Lueker et al.^72^ and the total pH scale were used. Seawater carbonate chemistry is summarized in Table 1.

**Table 1:** Mean (± SD) environmental variables for the control and reduced-pH treatments.

Treatment	n	Temperature (°C)	Salinity	DIC (μmol kg^−1^)	TA (μmol kg^−1^)	*p*CO_2_ (μatm)	pH_total_
Ambient pH	11	28.4 ± 0.3	37.9 ± 0.54	2,014 ± 35	2,317 ± 26	493 ± 48	7.96 ± 0.03
Reduced pH	8	28.4 ± 0.3	37.9 ± 0.48	2,195 ± 34	2,329 ± 32	1,253 ± 75	7.62 ± 0.02

Temperature, salinity, dissolved inorganic carbon (DIC), and total alkalinity (TA) were measured directly; *p*CO_2_ and pH_total_ were calculated in CO2SYS using constants from Lueker et al.^72^.

### Preparation of chemical-cue seawater and Y-maze experiments

The *Laurencia* spp. cue seawater was created by incubating 250 wet g of freshly collected *Laurencia* in 8 L of seawater (concentration of 31 g L^−1^) for 8 h in filtered natural seawater. The *Laurencia* was blotted dry before it was weighed. This concentration is within the range of those used in earlier studies that tested the effects of chemical cues on spiny lobsters^59,60^. The 8 L of cue seawater was transferred into an empty 20-L carboy for the experiment. The control consisted of 8 L of filtered seawater and was also stored in a 20-L carboy for experimentation. The *Laurencia* used to make the cue seawater was replaced with freshly harvested algae every 48 h. Cue seawater not used during the experimental trials was discarded and fresh batches of *Laurencia*. cue and control seawater were made daily.

**Figure 3:**
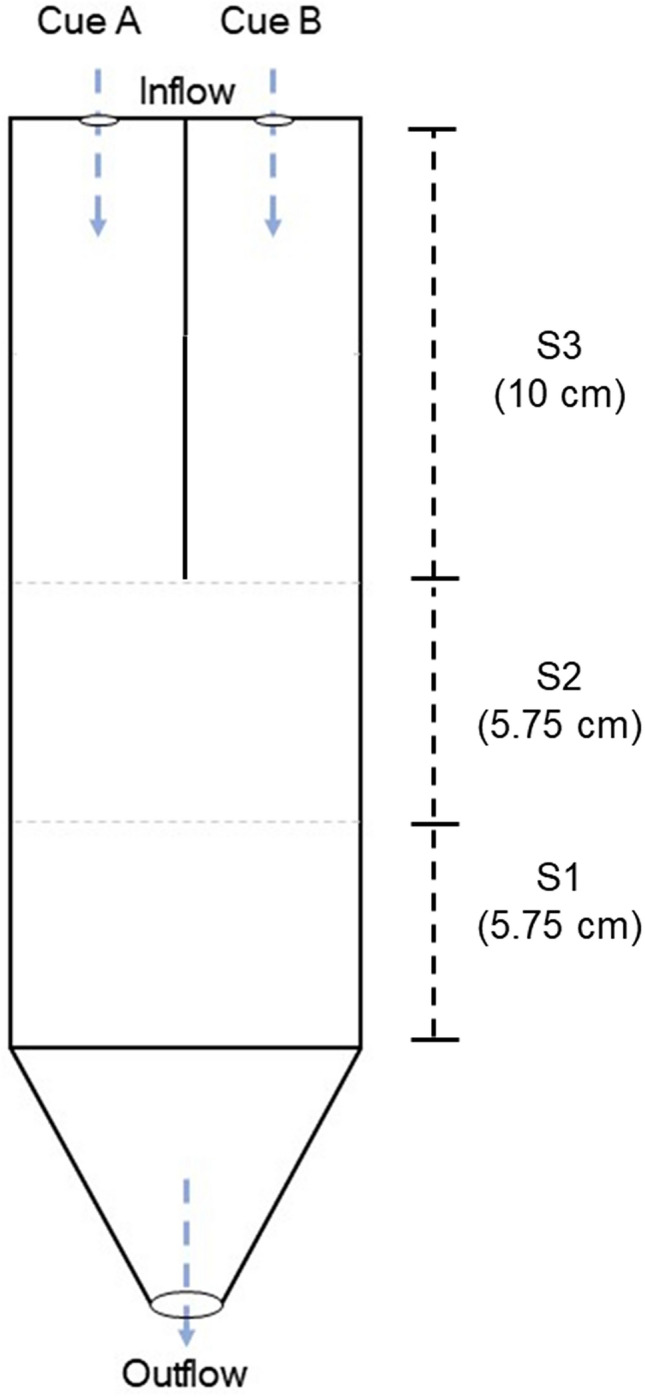
Depiction of the Y-maze chemical choice chamber used to test the responses of spiny lobster pueruli to *Laurencia* spp. chemical cues. The chamber was subdivided into sections for data collection and analysis (dotted horizontal lines; sections labelled S1–S3). Animal movements and the time spent within each section were monitored in each trial. Cue water was pumped in to either side A or B of the chamber and the side of the cue was randomized between trials. The dashed arrows (grey) represent directional seawater inflow and outflow.

Y-maze choice trials were conducted at night in a darkroom erected around a raceway in the CAOS experimental system and illuminated with far-red light (775 nm), which is beyond the visible spectrum for crustaceans^73,74^. The 8 L of *Laurencia* cue seawater and control seawater were transferred into separate 20-L carboys which were positioned on a stand so that they could be simultaneously pumped into the Y-maze (21.5 cm × 6 cm × 5.25 cm; Fig. 3) during experimentation. The Y-maze had opaque sides to prevent visual stimuli from affecting the orientation and behaviour of lobster pueruli. In addition, all trials were conducted at night and in a darkroom.

Delivery of the *Laurencia* cue seawater and control seawater to the Y-maze was achieved using two variable-speed peristaltic pumps (United States Plastic Corp.) connected to each reservoir carboy and that independently supplied each side of the Y-maze choice chamber with a precisely controlled seawater flow (120–125 mL min^−1^). The Y-maze was partitioned into three sections to characterize activity of a puerulus in the chamber. The first section was that in which a puerulus was introduced into the maze and was the section farthest from the source of the chemical cue (Fig. 3). The last section was closest to the source of the chemical cue. Dye tests were conducted independently of experimental trials to confirm that flow was unidirectional and that flow rates were the same on both sides of the Y-maze.

During each trial, an individual puerulus postlarva (n = 16 individuals per treatment condition) was introduced into the Y-maze and held within the first section with a mesh screen for a 2-min acclimation period, after which the mesh screen was removed and the location of the puerulus within the Y-maze (i.e., section number and cue side) was recorded every 5 s. Observations of the location of the puerulus in the chamber were recorded until either it had made an outright choice, defined as movement toward one side of the chamber and was beyond the partition (i.e., it was in the third section), or 5 min had lapsed. Each puerulus was used in only one trial. Between trials the Y-maze chamber was rinsed, first with freshwater, then with ambient natural seawater. The side of the chamber into which the cue was introduced was selected at random for each trial. The temperature and salinity of both the control and chemical-cue seawater were maintained within a narrow range throughout the study (Table 1).

### Data analysis

During each independent trial, we recorded the movement of pueruli by monitoring the proportion of time that each individual spent to move out of section one of the chamber. A puerulus was classified as being in a section if more than half of its body was in that section. We also recorded the side of the chamber to which each puerulus was attracted by monitoring whether individuals ended the trial in section three or beyond the Y-maze partition of the chamber.

Differences in the proportion of time spent in section one among treatments was determined using a Wilcoxon rank sum test. Differences in choice (cue vs. seawater control) by pueruli in the Y-maze were determined using a binomial test, in which the number of animals that entered the cue side of the chamber was compared to the number that did not enter the cue side of the chamber. If significant, the distribution observed during the chemical cue trial was compared statistically to that of the control using a G-test. All statistical analyses were performed using R v.3.6^71^.
